# Whole-exome sequencing enables rapid and prenatal diagnosis of inherited skin disorders

**DOI:** 10.1186/s12920-023-01628-2

**Published:** 2023-08-21

**Authors:** Zhu Xintong, Zhang Kexin, Wang Junwen, Wang Ziyi, Luo Na, Guo Hong

**Affiliations:** 1 Department of Medical Genetics, College of Basic Medical Science, Army Medical University, 30# Gaotanyan St., Shapingba District, Chongqing, 400038 P.R. China; 2 Department of Dermatology, Southwest Hospital, Army Medical University, 30# Gaotanyan St., Shapingba District, Chongqing, 400038 P.R. China

**Keywords:** Inherited skin disorders, Genetic diagnosis, Prenatal diagnosis, Whole exome sequencing (WES), Genetic counseling

## Abstract

**Background:**

Genodermatoses are a broad group of disorders with specific or non-specific skin-based phenotypes, most of which are monogenic disorders. However, it’s a great challenge to make a precise molecular diagnosis because of the clinical heterogeneity. The genetic and clinical heterogeneity brings great challenges for diagnosis in dermatology. The whole exome sequencing (WES) not only expedites the discovery of the genetic variations, but also contributes to genetic counselling and prenatal diagnosis.

**Materials and methods:**

Followed by the initial clinical and pathological diagnosis, genetic variations were identified by WES. The pathogenicity of the copy number variations (CNVs) and single-nucleotide variants (SNVs) were evaluated according to ACMG guidelines. Candidate pathogenic SNVs were confirmed by Sanger sequencing in the proband and the family members.

**Results:**

Totally 25 cases were recruited. Nine novel variations, including c.5546G > C and c.1457delC in *NF1*, c.6110G > T in *COL7A1*, c.2127delG in *TSC1*, c.1445 C > A and c.1265G > A in *TYR*, Xp22.31 deletion in *STS*, c.908 C > T in *ATP2A2*, c.1371insC in *IKBKG*, and nine known ones were identified in 16 cases (64%). Prenatal diagnosis was applied in 6 pregnant women by amniocentesis, two of whom carried positive findings.

**Conclusions:**

Our findings highlighted the value of WES as a first-tier genetic test in determining the molecular diagnosis. We also discovered the distribution of genodermatoses in this district, which provided a novel clinical dataset for dermatologists.

**Supplementary Information:**

The online version contains supplementary material available at 10.1186/s12920-023-01628-2.

## Introduction

Genodermatoses are a broad group of disorders with specific or non-specific skin-based phenotypes, most of which are monogenic disorders [1]. Currently, there are more than 1000 monogenic skin disorders, which could be mainly classified into categories according to the clinical features: disorders of cornification, epidermolysis and blistering disorders, hypo- or hyperpigmentation, connective tissue defects and tumor predisposition [1, 2]. The clinical diagnosis of genodermatoses could be based on the special clinical manifestations and dermatopathological features. However, it’s a great challenge to make a precise molecular diagnosis because of the clinical heterogeneity. It brings great difficulties for the prevention and genetic counseling for the hereditary skin diseases.

Over the last 35 years, the molecular basis of inherited skin diseases has been extensively elucidated, with some initial discoveries driven by candidate gene approaches [1, 3]. Sanger sequencing is always the gold standard method to detect the pathogenic single nucleotide variants (SNVs), even the low throughput limits its use. More importantly, this approach depends on the known genes that have been identified. The introduction of next-generation sequencing (NGS) technology has greatly accelerated and improved diagnostic accuracy for genodermatoses [3–5]. The advent of targeted enrichment of specific genes of interest or whole-exome sequencing (WES), is now becoming available to perform a more efficient screening of such highly heterogeneous disorders. Targeted NGS strategies focus on multigene panels were used in molecular diagnosis with its low cost and high accuracy [6, 7]. However, as the updates of the disease genes and the falling costs of NGS, multigene panels were gradually replaced by WES due to the lower positive rate and coverage [8]. In the past decade, NGS was used to discover nearly 200 novel genes contributing to inherited skin disease. Approximately 90% of the discoveries were made by using WES.

In the present study, we investigated 25 Chinese patients with various forms of genodermatoses at clinical and molecular level. Combining clinical and pathological phenotypes, the patients were classified into four categories: keratosis, epidermolysis, parachromatosis and tumor predisposition. WES was used to identify the diseases causing sequence variants for these probands. Candidate pathogenic SNVs were confirmed by Sanger sequencing in the family members. We accurately diagnosed 16 patients and determined 9 known variants and 9 novel pathogenic variants which are hypothesized to cause the corresponding inherited skin diseases. In six families, we successfully applied WES to the prenatal diagnosis of 6 pregnant women. Thus, our research confirms that the clinical application of WES is an effective diagnostic strategy that speeds up the identification of etiology and improves prenatal diagnosis in an accurate way.

## Materials and methods

### Research subjects

The study was approved by the Ethics Committee of Southwest Hospital of Army Medical University (Chongqing, China). The written informed consent was obtained from all the participants. All the unrelated 25 patients with hereditary skin diseases were identified and enrolled at Southwest Hospital and Xinqiao Hospital of Army Medical University from January 2018 to December 2020. The probands and their families have undergone physical examinations and health assessments in the Dermatology Department. Peripheral blood samples were collected from the patients, their family members, and healthy controls. At 16–18 weeks of gestation, fetal amniotic fluid was obtained by amniocentesis under ultrasound monitoring. DNA was extracted from each blood sample and amniotic fluid by using Wizard Genomic DNA Purification Kit (Promega, USA) according to the protocol. The quantity and quality of DNA was determined by using a NANODROP 1000 (Thermo Fisher Scientific, Waltham, MA, USA).

### Whole exome sequencing

The genomic DNA of the patients was fragmented to generate 200–300 bp fragments using Covaris S2 (Covaris, Massachusetts, USA) according to the manufacturer’s instructions. Paired-end libraries were prepared following the Illumina library preparation protocol. The exome was captured using the SureSelect Human All Exons Plus kit (Agilent). Paired-end sequencing was carried out on an Illumina HiSeq 2000 sequencer (Illumina, San Diego, CA, USA) with a read length of 2 × 100 bp, average depth of at least 50×, coverage at least 98.5%, fraction of target covered over 20× at least 90% for each sample. Raw image files were processed by the Illumina Pipeline for base calling using default parameters. Primary data was in fastq format after image analysis and base calling was conducted using the Illumina Pipeline. The data was filtered to generate “clean reads” by removing adapters and low-quality reads. The raw results were analysed by using a standard pipeline that utilized published algorithms in a sequential manner. Sequencing reads were mapped to the reference human genome version hg19 (http://genome.ucsc.edu/). Variant analysis was performed using SOAPsnp software (V1.05) and Samtools (V1.5) for SNPs and indels, respectively. All SNPs were identified by using the NCBI dbSNP, HapMap,1000 human genome dataset (http://www.1000genomes.org/), the Exome Aggregation Consortium (ExAC), the Chinese Millionome Database (CMDB) (https://db.cngb.org/cmdb/), local database of BGI (Shenzhen, China) and the database of Chigene Translational Medical Research Center (Beijing, China).

### Sanger sequencing and functional prediction of variations

Sanger sequencing was used to confirm the results of WES or WGS and to perform prenatal diagnosis in pregnant women. The sanger sequencing was performed using a previous protocol. The software Primer3 was used to design the primers. The primer pairs for PCR are available upon request. PCR reactions were carried out under standard conditions as follows: one cycle for 5 min at 94 °C followed by 30 cycles at 94 °C for 30 s, 60 °C for 30 s, 72 °C for 30 S, and one last cycle at 72 °C for 5 min. The purified PCR products were directly sequenced in both forward and reverse directions by ABI 3130 genetic analyzer with the Big Dye Terminator Cycle sequencing reaction kit (Applied Biosystems, Foster City, CA, USA) according to the manufacturer’s instructions. Sequences were analyzed using the vector NTI 11.0 software package. The sequence results were compared with the updated consensus sequence.

Several online prediction softwares were used to predict the functional significance of the mutations, including PolyPhen 2 (http://genetics.bwh.harvard.edu/pph2), SIFT (http://sift.jcvi.org), MutationTaster (http://www.mutationtaster.org/) and I-Mutant v2.0 (http://folding.biofold.org/i-mutant/i-mutant2.0.html). Combined Annotation Dependent Depletion (CADD, https://cadd.gs.washington.edu/) is a tool for scoring the damaging effects of single nucleotide variants as well as insertion/deletions variants in the human genome. The pathogenicity of the SNVs were evaluated according to American College of Medical Genetics guidelines (ACMG) guideline [9].

## Results

### General information of the cases

A total of 25 cases with hereditary skin disorders were collected in the study, including 14 males (56%) and 11 females (44%). Most of the cases (92%) were noticed and clinically diagnosed through appearance of the lesions, laboratory examination and/or pathological biopsy, in which 12 cases (48%) with positive family history. The molecular diagnosis depended on the genetic screening by WES. In most cases, the probands were born with the disease or developed the disease in early childhood. However, the diagnosis was made in a later age, ranging from 5 to 58 years old. In our study, the cases could be grouped into four broad categories, including tumor predisposition, disorder of cornification, epidermolysis and abnormal pigmentation. Among all the 25 cases, tumor predisposition was the most common type (9/25, 36%), including tuberous sclerosis complex and neurofibromatosis. The second is inherited cornification disorders (7/25, 28%), consisting of palmoplantar keratosis, ichthyosis vulgaris, keratosis follicularis and pachyonychia congenita. The third is abnormal pigmentation (5/25, 20%), including albinism, incontinentia pigmenti and becker nevus syndrome. There are 4 cases with epidermolysis (4/25, 16%). As shown in Figure S1, the distribution of these inherited skin disease cases were: neurofibromatosis (5/25, 20%), epidermolysis bullosa (4/25, 16%), tuberous sclerosis complex (4/25, 16%), albinism (3/25, 12%), ichthyosis vulgaris (2/25, 8%) and keratosis follicularis (2/25, 8%).

### Cases with positive genetic findings

Within the 25 cases, 16 patients (64%) were accurately diagnosed with inherited skin diseases by WES. According to the international guidelines of ACMG [9], nine known variations and nine novel ones were classified as pathogenic/likely pathogenic, including c.5546G > C and c.1457delC in *NF1*, c.6110G > T in *COL7A1*, c.2127delG in *TSC1*, c.1445 C > A and c.1265G > A in *TYR*, Xp22.31 deletion in *STS*, c.908 C > T in *ATP2A2*, c.1371insC in *IKBKG* (Table 1). In which, 12 variations were inherited from the parents and 1 was de novo. All kinds of SNVs were present, including 7 missense variations, 5 deletions, 2 insertions and 4 nonsense mutations. There were 2 compound heterozygous variations, 11 heterozygous, 2 homozygous and 1 hemizygous. Dominant or recessive inheritance way could both be found, with the dominant in the majority. Only two cases were X-linked, one X-linked dominant and one X-linked recessive. All these mutations were not found in the normal controls.

**Table 1:** SNVs identified by WES in 25 patients with inherited skin disorders

Case	Age	Sex	Diagnosis	Gene	Nucleotide change	Protein change	mutation	Het/Hom	Inheritance	PE	Reported
#1	38	F	Neurofibromatosis	*NF1*	c.5546G>C	p.R1849P	missense	Het	AD	P	this study
#2	31	F	Neurofibromatosis	*NF1*	c.5242 C>T	p.R1748X	nonsense	Het	AD	P	reported
#3	28	F	Neurofibromatosis	*NF1*	c.1457delC	p.T486Kfs*12	frameshift	Het	DN	P	this study
#4	36	M	Neurofibromatosis	negative	NA	NA	NA	NA	Un	NA	NA
#5	27	F	Neurofibromatosis	negative	NA	NA	NA	NA	AD	NA	NA
#6	18	M	Epidermolysis bullosa	*COL7A1*	c.6110G>T	p.G2037V	missense	Het	AD	P	this study
#7	58	F	Epidermolysis bullosa	negative	NA	NA	NA	NA	AD	NA	NA
#8	13	F	Epidermolysis bullosa	negative	NA	NA	NA	NA	Un	NA	NA
#9	29	F	Epidermolysis bullosa	negative	NA	NA	NA	NA	Un	NA	NA
#10	34	F	Tuberous sclerosis complex	*TSC1*	c.2127delG	p.R709Sfs*15	frameshift	Het	AD	P	this study
#11	27	M	Tuberous sclerosis complex	*TSC1*	c.2690delT	p.F897Sfs*	frameshift	Het	AD	P	reported
#12	7	F	Tuberous sclerosis complex	*TSC2*	c.3206_3207delTG	p.V1069fs)	frameshift	Het	AD	P	reported
#13	23	M	Tuberous sclerosis complex	negative	NA	NA	NA	NA	Un	NA	NA
#14	30	M	Albinism	*TYR*	c.230_232dupGGG	p.R77_E78insG	duplication	C-Het	AR	P LP	reported this study
					c.1445 C>A	p.A482E	missense				
#15	5	F	Albinism	*TYR*	c.346 C>T c.1265G>A	p.R116X p.R422Q	nonsense missense	C-Het	AR	P P	reported this study
#16	32	M	Albinism	*TYR*	c.832 C>T	p.R278X	nonsense	Hom	AR	P	reported
#17	33	M	Ichthyosis vulgaris	negative	NA	NA	NA	NA	AD	NA	NA
#18	24	M	Ichthyosis vulgaris	*STS*	Xp22.31 deletion	na	deletion	Hem	XR	P	this study
#19	28	F	Keratosis follicularis	*ATP2A2*	c.908 C>T	p.A303V	substitution	Het	AD	P	this study
#20	17	M	Keratosis follicularis	*ATP2A2*	c.2381T>A	p.V794D	missense	Het	AD	P	reported
#21	30	F	Pachyonychia congenita	*KRT16*	c.383T>C	p.L128P	missense	Het	AD	P	reported
#22	32	F	Incontinentia pigmenti	*IKBKG*	c.1371insC	p.E458Rfs*5	frameshift	Het	XD	P	this study
#23	25	M	Palmoplantar keratosis	*SERPINB7*	c.796 C>T	p.R266X	nonsense	Hom	AR	P	reported
#24	43	M	Becker nevus syndrome	negative	NA	NA	NA	NA	Un	NA	NA
#25	35	F	Steatocystoma multiplex	negative	NA	NA	NA	NA	Un	NA	NA

Sex: M, male, F, female. Het: Heterozygosity/Heterozygous C-Het, Compound heterozygous, Hem, Hemizygous. Inheritance: DN, de novo, AD, Autosomal dominant, AR, Autosomal recessive, XD, X-linked dominant, XR, X-linked recessive, Un, Untested. Pathogenicity evaluation (PE), P, Pathogenic, LP, Likely pathogenic, VUS, Uncertain clinical significance, NA, Not available

**Fig. 1:**
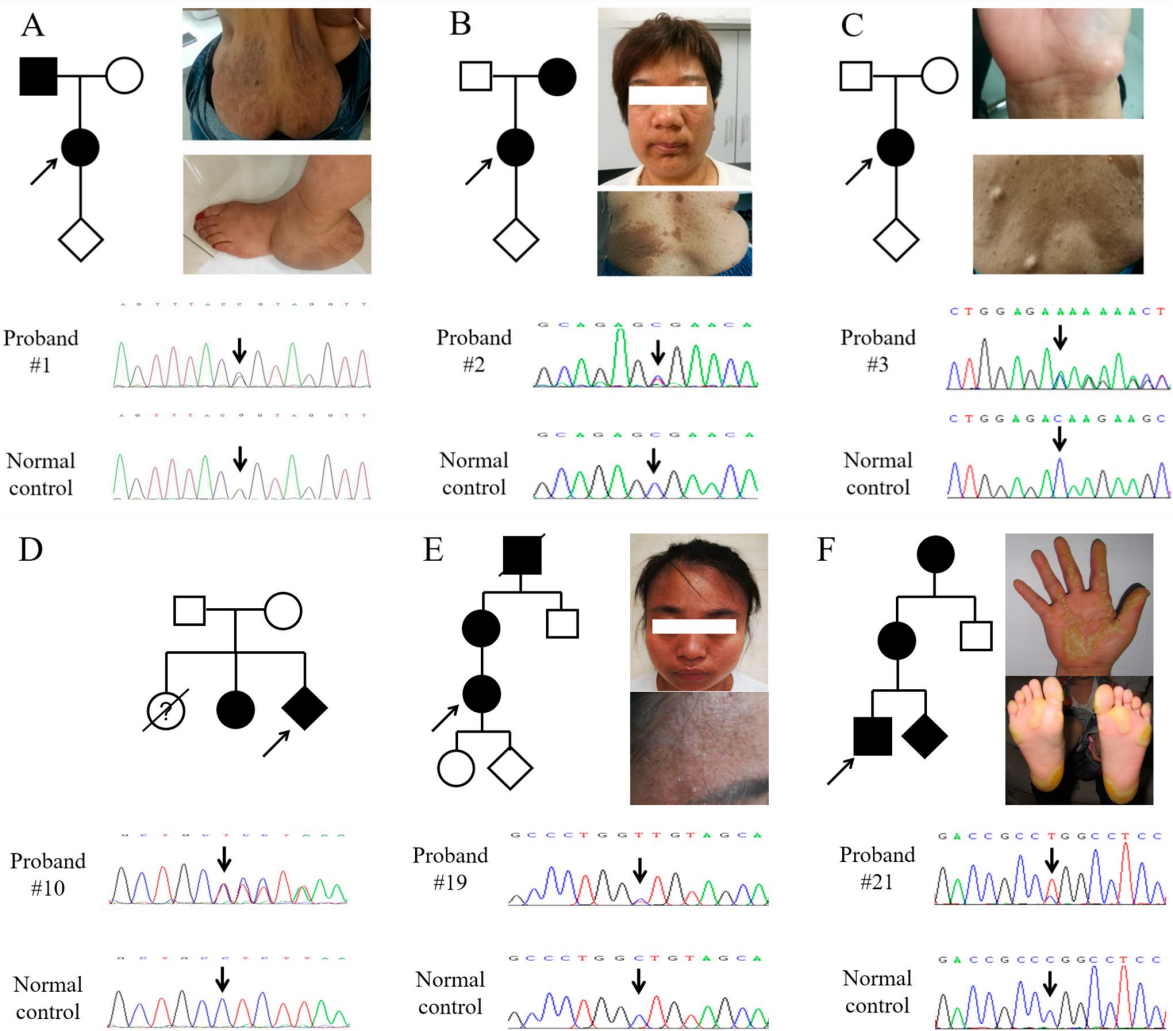
Prenatal diagnosis was applied in six pregnant female probands with molecular diagnosis. (**A**) Proband #1 showed huge fibromas on the back and left foot, with a heterozygous mutation in *NF1* (c.5546G > C, p.R1849P) inherited from the father. (**B**) Café-au-lait spots presented on the trunk and neck for proband #2, with a heterozygous mutation in *NF1* (c.5242 C > T, p.R1748X) inherited from the mother. (**C**) Multiple café-au-lait spots were found on the back and hand in proband #3, who carried a heterozygous de novo mutation in *NF1* (c.1457delC, p.T486Kfs*12). (**D**) The asymptomatic case (#10) gave birth to a 4 years old girl with seizure. A heterozygous mutation in *TSC1* (c.2127delG, p.R709Sfs*15) was identified in the mother, the daughter and the fetus. (**E**) Proband #19 showed skin itching and rash mainly on the head and face, with a heterozygous mutation in *ATP2A2* (c.908 C > T, p.A303V) from her mother. (**F**) The patients from the autosomal dominant family showed mainly thick nails and palmoplantar keratosis. A heterozygous mutation in *KRT16* (c.383T > C, p.L128P) was identified in all the patients and the fetus

**Fig. 2:**
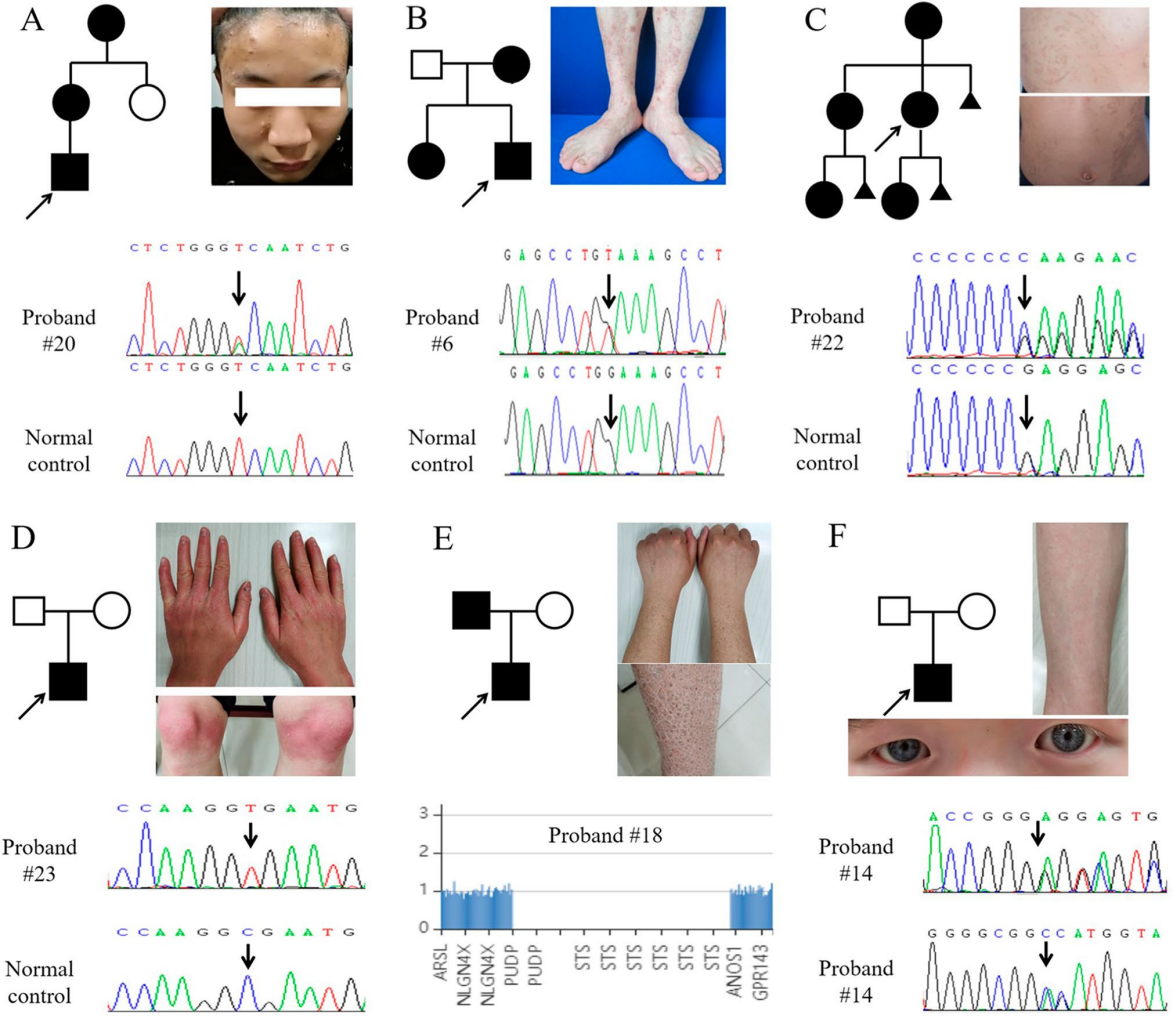
The other cases with positive genetic findings. (**A**) Skin pruritus and rash mainly affected the head, face and trunk of proband #20, with a heterozygous missense mutation in *ATP2A2* (c.2381T > A, p.V794D). (**B**) A heterozygous missense mutation in *COL7A1* (c.6110G > T, p.G2037V) was identified in proband #6, presented with blisters on the extensor side of the extremities and positive Nissl‘s sign. (**C**) Proband #22 showed linear-distributed pigmented macula on the trunk, with a heterozygous framshift mutation in *IKBKG* (c.1371insC, p.E458Rfs*5) inherited in an X-linked dominant way. (**D**) Proband #23 showed large erythema on his hands, feet, bilateral knee joints and elbow joints, accompanied by keratinization and peeling. A homozygous nonsense mutation in *SERPINB7* (c.796 C > T, p.R266X) was inherited from non-consanguineous parents. (**E**) Clinical features of proband #18 showed dry skin with scaly desquamation involving the limbs and trunk since birth. A hemizygous deletion of Xp22.31 involving STS was identified by WES. (**F**) The proband #14, with typical ocular skin albinism, carried a compound heterozygous mutation in *TYR* (c.230_232dupGGG, p.R77_E78insG; c.1445 C > A, p.A482E)

In the 16 cases with a precise genetic diagnosis (Figs. 1 and 2), neurofibromatosis (3, 18.75%), tuberous sclerosis complex (3, 18.75%), albinism (3, 18.75%), keratosis follicularis (2, 12.5%), epidermolysis bullosa (1, 6.25%), ichthyosis vulgaris (1, 6.25%), pachyonychia congenita (1, 6.25%), incontinentia pigmenti (1, 6.25%) and palmoplantar keratosis (1, 6.25%) were identified. In which, neurofibromatosis, tuberous sclerosis complex and albinism were the top ones, which added up to 56.25% of all 16 cases. Most of the disorders were inherited in an autosomal dominant way (56.25%).

### Prenatal diagnosis

Of all the 25 cases, six were pregnant women who had not been definitively diagnosed. Four of them had a family history, with the same clinical phenotypes in their parents. One case (#3) was diagnosed to be neurofibromatosis clinically without a family history. The other asymptomatic case (#10) gave birth to a 4 years old girl with seizure who didn’t get a molecular diagnosis previously. Case #10 became pregnant again and a cardiac rhabdomyoma was detected during the prenatal ultrasound at the 23rd week of gestation. For all 6 pregnant women and the patients inside the families, WES was applicated first. As shown in Fig. 1, all 6 probands got a definitive molecular diagnosis, including 3 neurofibromatosis, 1 tuberous sclerosis complex, 1 keratosis follicularis and 1 pachyonychia congenita. After that, we performed prenatal diagnosis of the fetus through amniocentesis and Sanger sequencing. Of all the six amniotic fluid cases, two were found to inherit mutations from pregnant women. The pregnant women received prenatal genetic counseling based on the genetic diagnosis. For case #10, the fetus was 36 weeks old when the genetic diagnosis was obtained, so they decided to continue the pregnancy.

### The cases without molecular diagnosis

**Fig. 3:**
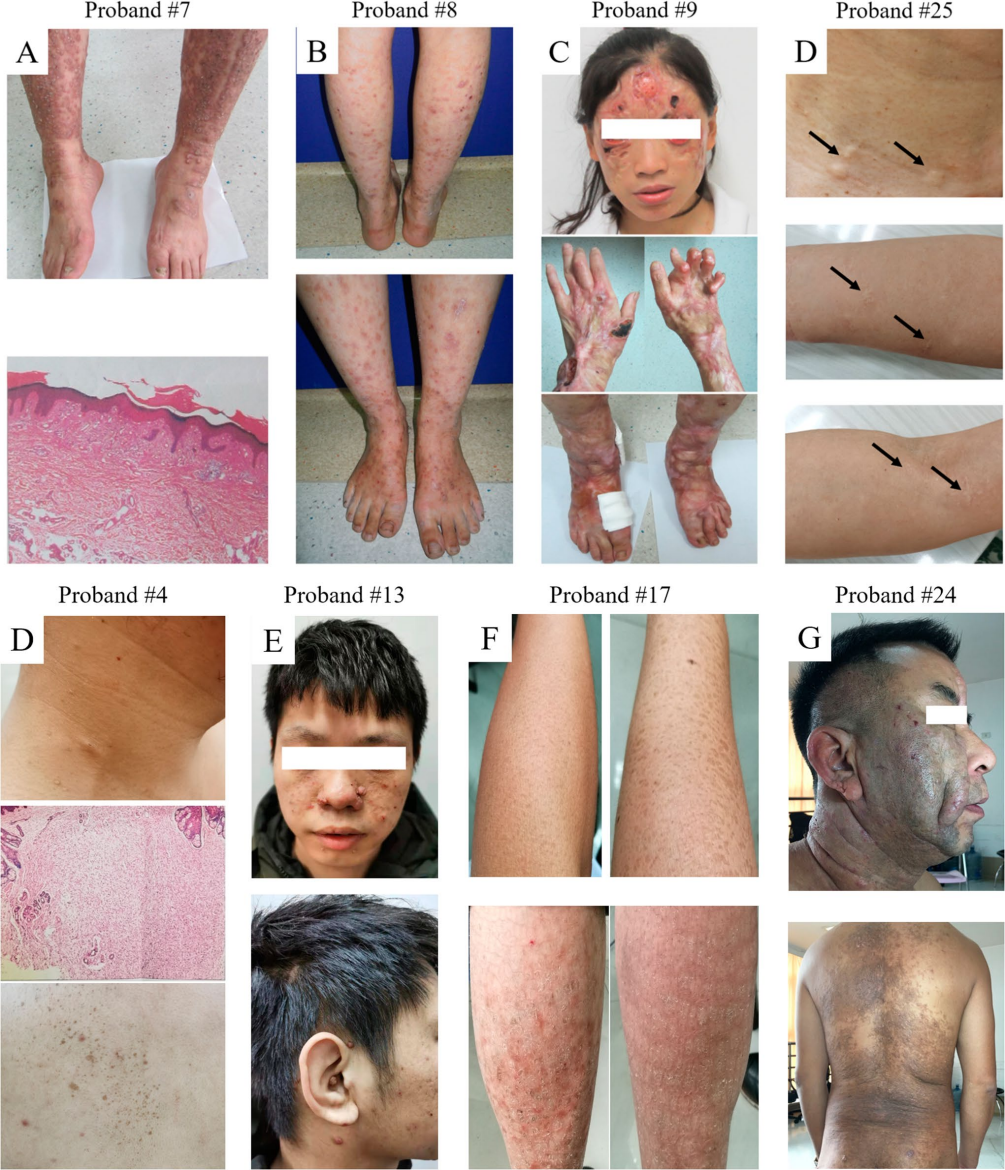
The cases without molecular diagnosis. (**A-C**). The three patients were clinically considered as epidermolysis bullosa. Blisters mainly on the extensor sides of the extremities. Scars or atrophy left after the skin lesions healed, which affected the function of the joints. (**D**) Proband #25 manifested as multiple cystic nodules on the neck, limbs and trunk. Steatocystoma multiplex is clinically considered. (**E**) The clinical features of Proband #4 showing café-au-lait spots on his trunk and small fibromas on his neck. (**F**) Proband #13 is characterized by multiple tough yellow telangiectatic papules on the face. (**G**) Proband #17 from a 3-generation autosomal dominant family with ichthyosis vulgaris, presented dry skin with scaly desquamation on his extremities since birth. (**H**) The clinical manifestation of proband #24 was dark brown patches on the face and back. Follicular papules and short bristles could be seen on the surface of the skin lesions. Becker’s nevus syndrome is considered

In all the 25 cases, there were still 9 cases without the molecular diagnosis. Combined with the typical clinical features and pathological changes, the 9 patients were clinically diagnosed with inherited skin diseases. 2 cases were considered neurofibromatosis and 3 cases were related to epidermolysis bullosa (Fig. 3). The remaining 4 cases were initially diagnosed as tuberous sclerosis complex, ichthyosis vulgaris, Becker nevus syndrome and steatocystoma multiplex respectively (Fig. 3). Case #17 from a 3-generation autosomal dominant family, had typical phenotype of ichthyosis vulgaris (IV). No mutation was identified in the known IV genes, such as *FLG*. For case #24, the patient was diagnosed to be Becker nevus syndrome, which characterized by the presence of a vast unilateral Becker nevus on the face, the back and the waist. The epidermal component of the nevus consisted of slight acanthosis and hyperpigmentation of basal cells. Case #25 was suspected to be steatocystoma multiplex and experienced several cystectomies. There were thousands of round or oval cystic tumors widely distributed on the neck, limbs and trunk. For all the 9 cases, no pathogenic mutations were found by WES. These cases require further study by other techniques, such as whole genome sequencing (WGS) or whole transcriptome sequencing (WTS).

## Discussion

Genodermatoses encompass a range of inheritable rare skin disorders with multisystem involvement, characterized by heterogeneous clinical course and prognosis. Today, more than 500 distinct mendelian skin disorders were reported in OMIM (https://omim.org/). They are subdivided into phenotypic categories such as blistering disorders, abnormal cornifications, abnormal pigmentation disorders, connective tissue diseases, ectodermal dysplasias, vascular/immune diseases, hair/nails disorders and syndromes associated with tumor predisposition [1, 2]. Several types can be life threatening whereas some are benign, thus accurate and rapid diagnosis is warranted. The first step in identifying a genodermatosis is a complete dermatologic examination, including the skin, mucosal, hair, nails and teeth [10]. To help establish the diagnosis, a thorough personal/family history, together with previous histologic or radiographic results should also be collected. Recognizing many genodermatoses sometimes is straightforward, as many conditions are visually recognizable, such as neurofibromatosis [11, 12]. The majority of our cases were also clinically diagnosed directly, with which 48% of the cases with positive family history. Here, in the 25 Chinese patients, tumor predisposition was the most common type, including 5 neurofibromatosis and 4 tuberous sclerosis complexes. NFs and TSCs are also one of the most common genodermatoses, which need long-term follow-up. Other common disease types included disorders of cornification, abnormal pigmentation and epidermolysis, which are basically consistent with the results of previous studies [3, 10].

Molecular diagnosis aimed at the identification of mutations in affected individuals helps to confirm the diagnosis and determine the genotype-phenotype correlations. Traditional genetic testing involves the analysis of a particular gene through Sanger sequencing, which is low throughput and time-consuming. In recent years, there are more than 1000 genes associated with a cutaneous phenotype [2, 3]. The traditional methods can no longer meet the needs of molecular diagnosis. High-throughput sequencing provides an expedient and cost-effective platform to identify pathogenic mutations. Customized multigene panels enable a large group of known genes associated with a particular phenotype to be evaluated in a cost-effective manner [6, 7]. For a long time, most of the patients refused the molecular tests due to poor rates of insurance coverage. The prevalence of clinical use of NGS is still low in this district, so a paucity of data exists. Here in the study of the 25 patients, we applied WES to identify the disease-causing variants, which were verified by Sanger sequencing subsequently. 16 patients were accurately diagnosed by WES, including nine known variations and nine novel ones. In which, 12 variations were inherited from the parents and 1 was de novo. Consistent with earlier studies, most patients harbored private mutations, along with a diverse clinical presentation [3, 13]. According to our data, molecular testing was diagnostic in 16 individuals (64%), which was higher than the conventional WES diagnostic rate ranging between 20% and 50% [3, 13, 14]. That might attribute to the that nearly 48% of the cases have positive family histories. A clear clinical diagnosis of these cases might be another important reason. In addition, based on the results of WES, we disclose the trend of inherited skin diseases in this region, which provides a clinical guide for dermatologists. Taken together, as an efficient, economically competitive, and frontline test, WES should be routinely ordered in the out-patient clinic for genodermatoses [3, 14].

However, it is worth noting that there are still some obstacles. As noted in several diagnostic studies previously, standardized phenotypic data and integrated variation deciphering process were crucial for the clinical application of WES [3, 15, 16]. Methods of processing the huge amounts of NGS data are still being optimized. The interface between genetics and dermatology has updated the classification systems that integrate clinical and molecular data. Therefore, as noted earlier, multidisciplinary clinics devoted to genodermatoses can be a good solution [17]. The expertise of dermatology, medical genetics, surgery and obstetrics were combined to derive genetic counseling, prenatal diagnosis and disease treatment. The patients and their families could obtain helpful instructions and support.

Even it’s a time-intensive process, some of our cases were handled in multidisciplinary clinics. Before performing genetic analysis, the potential benefits, limitations, and risks were carefully discussed with the affected individuals and families [18, 19]. Here we performed precise genetic counselling and prenatal diagnosis for six pregnant women with the molecular diagnostic results. Among them, the fetus of the case #10, having an abnormal heart structure found by ultrasound, brought the attention of the obstetrician. The mutation was identified in the asymptomatic mother and her daughter with seizure firstly by WES. Then prenatal diagnosis was performed in the fetus by Sanger sequencing. Finally, put all the pieces together, the pregnant woman decided to continue the pregnancy at 36 weeks of gestation. Similarly, the fetus of the proband 21 was diagnosed with pachyonychia congenita after Sanger sequencing of amniotic fluid. However, the pathogenic variants were not found in the other four fetuses during the prenatal diagnosis. The postnatal follow-up confirmed these findings. Based on our practice, the results of prenatal diagnosis might help patients with early intervention in the fetus, newborn and even childhood [18].

In all the 25 cases, there were still 9 cases without molecular diagnosis for the following reasons. Inherited skin diseases are often variable in their clinical manifestations [2]. Patients may have clinically recognizable disorders that still lack a genetic basis. For the case #24, the patient was diagnosed to be Becker nevus syndrome, which has been reported in OMIM (604,919), without any known disease-causing genes identified. No mutation was found in KRT17 in the patient #25, with typical steatocystoma multiplex. Both of the two patients had no family history, so it’s difficult to find the chromosomal location of the diseases. This might also be concerned with the limitations of WES, which was not suited to detecting noncoding variants and structural variants [3, 20]. WES may also miss critical exons or the chimeric mutations in disease-associated genes [3, 21]. For case #17, the patients from the 3-generation autosomal dominant family had a typical phenotype of ichthyosis vulgaris, but no mutation was identified in the known genes, such as *FLG*. Consideration must also be given to the copy-number variant or common mutations [21]. The clinical application of WES still requires efforts to close some of the shortfalls, such as increasing accuracy rates and decreasing interpretation inaccuracy [4, 9, 15]. Other techniques are emerging to be utilized as complementary approaches. Full-coverage genomic studies like WGS will play an important role, assisted by other techniques such as the epigenome, transcriptome and proteome [22, 23]. These techniques may explore broader phenotypic spectrums, novel disease-causing genes and modifiers.

## Conclusions

In summary, we described the disease phenotypes and explored the genetic etiology by WES in a small cohort of 25 Chinese genodermatoses patients. The group exhibited a wide spectrum of clinical variability ranging from a mild predominantly acral blistering phenotype to a severe generalized phenotype. We accurately diagnosed 16 patients and discovered 9 known variants and 9 novel pathogenic variants. In six families, we successfully applied WES on the prenatal diagnosis for 6 pregnant women. These data further extended the variation spectrum of genodermatoses in this district. Our research further confirms that the clinical application of WES is an effective diagnostic strategy which speeds up the identification of etiology and improves prenatal diagnosis in an accurate way.

### Electronic supplementary material

Below is the link to the electronic supplementary material.

Supplementary Material 1

## Data Availability

The datasets used and/or analysed during the current study are available from the corresponding author on reasonable request. All these mutations have been submitted to the ClinVar database (https://submit.ncbi.nlm.nih.gov/clinvar/), the submission numbers of the date are SUB13573777, SUB13573766, SUB13573757, SUB13573743, SUB13573735, SUB13573690, SUB13573677, SUB13573569.
