# Supplementary material for: Whole-exome sequencing enables rapid and prenatal diagnosis of inherited skin disorders
Source: BMC Med Genomics. 2023 Aug 21;16:193. doi: 10.1186/s12920-023-01628-2 (PMC10440863; doi:10.1186/s12920-023-01628-2)
Supplement: Supplementary file 1 — Supplementary Material 1 [file 12920_2023_1628_MOESM1_ESM.docx]

**Supplementary File**

**
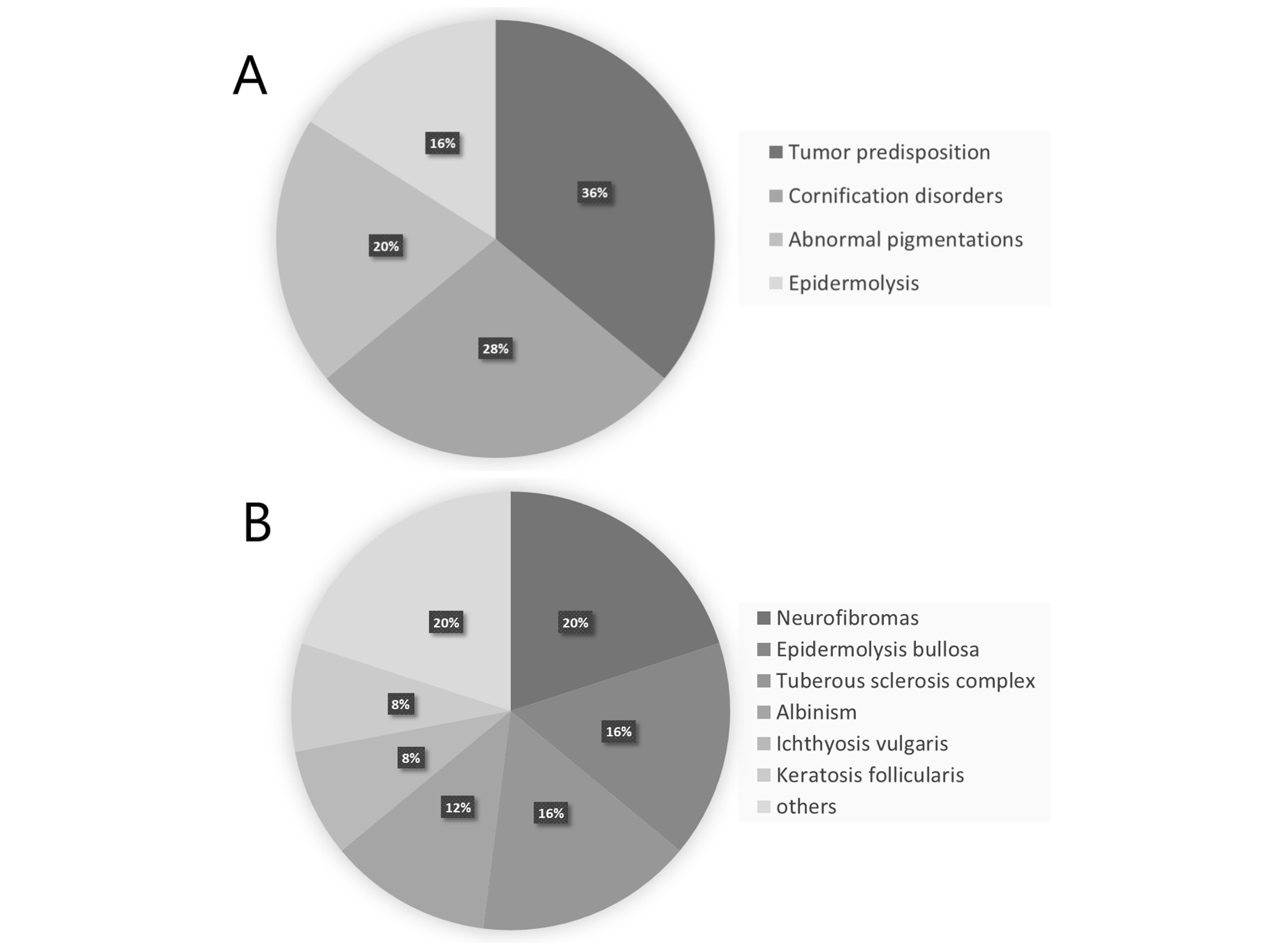
**

**Figure S1. The classification and distribution of inherited skin diseases in this cohort from Chongqing.** (A) The patients were classified into four categories: tumor predisposition (9/25, 36%), cornification disorders (7/25, 28%), abnormal pigmentations (5/25, 20%) and epidermolysis (4/25, 16%). (B) The distribution of the diagnosed cases with inherited skin diseases. The 3 relatively most common monogenic skin diseases were neurofibromatosis (5/25, 20%), epidermolysis bullosa (4/25, 16%) and tuberous sclerosis complex (4/25, 16%), followed by albinism (3/25, 12%), ichthyosis vulgaris (2/25, 8%) and keratosis follicularis (2/25, 8%).
